# Protective role of school climate for impacts of COVID-19 on depressive symptoms and psychotic experiences among adolescents: a population-based cohort study

**DOI:** 10.1017/S0033291724003192

**Published:** 2024-12

**Authors:** Satoshi Yamaguchi, Jordan DeVylder, Syudo Yamasaki, Shuntaro Ando, Mitsuhiro Miyashita, Mariko Hosozawa, Kaori Baba, Junko Niimura, Naomi Nakajima, Satoshi Usami, Kiyoto Kasai, Mariko Hiraiwa-Hasegawa, Atsushi Nishida

**Affiliations:** 1 Unit for Mental Health Promotion, Research Center for Social Science & Medicine, Tokyo Metropolitan Institute of Medical Science, 2-1-6 Kamikitazawa, Setagaya-ku, Tokyo 156-8506, Japan; 2 Silver School of Social Work, New York University, Washington Square North, New York, NY, 10003-6654, USA; 3 Department of Neuropsychiatry, Graduate School of Medicine, University of Tokyo, 7-3-1 Hongo, Bunkyo-ku, Tokyo 113-0033, Japan; 4 Institute for Global Health Policy Research, Bureau of International Health Cooperation, National Center for Global Health and Medicine, 1-21-1 Toyama, Shinjuku-ku, Tokyo, 162-8655, Japan; 5 Division of Educational Psychology, Graduate School of Education, University of Tokyo, 7-3-1 Hongo, Bunkyo-ku, Tokyo 113-0033, Japan; 6 Center for Research and Development on Transition from Secondary to Higher Education, University of Tokyo, 7-3-1 Hongo, Bunkyo-ku, Tokyo 113-0033, Japan; 7 International Research Center for Neurointelligence (WPI-IRCN), University of Tokyo Institutes for Advanced Study (UTIAS), 7-3-1 Hongo, Bunkyo-ku, Tokyo 113-0033, Japan

**Keywords:** adolescent, COVID-19, depressive symptoms, population-based cohort, psychotic experiences, school climate

## Abstract

**Background:**

Schools are central places for adolescent social lives, which is a major factor greatly affecting adolescent mental health; school climate (i.e. quality of the school social environments) can be a proximal social determinant for adolescent mental health. Supportive school environments may serve as a protective factor during crises like COVID-19, which disrupt social lives and worsen adolescent mental health. This is the first study examining whether the pandemic effects differed based on the levels of school climate on depressive symptoms (DS) and psychotic experiences (PEs) among adolescents.

**Methods:**

School climate (score range: 0–28), DS (0–26), and PEs (0–5) were self-reported in a population-based cohort (Tokyo Teen Cohort; *N* = 3171) at four timepoints (10y, 12y, 14y, and 16y) before and during COVID-19. COVID-19 occurred midway through the 16y survey, allowing us to examine its impact and interaction effect with school climate while accounting for within-person changes over time using mixed-effects models.

**Results:**

Significant interaction effects were found on DS (unstandardized coefficient [*B*] = −0.166, 95% confidence interval [CI] −0.225 to −0.107) and PEs (*B* = −0.020, 95% CI −0.028 to −0.012). The pandemic effects were not significant for adolescents with high school climate scores (around the 80th percentile or higher), although the pandemic significantly worsened these outcomes among the overall sample.

**Conclusions:**

The negative mental health effects of the pandemic were significantly mitigated among adolescents experiencing a supportive school climate. A positive school climate can protect adolescent mental health during challenging social conditions, such as pandemics.

## Introduction

Schools play a central role in adolescent social lives, which is closely related to adolescent mental health (Shinde et al., 2018, 2020). Adolescent self-rated school climate is an indicator of the quality of social environments in schools (e.g. interpersonal relationships among students with teachers/peers, and participation in school activities/decisions) (Shinde et al., 2018), which, therefore, can be considered a proximal social determinant for adolescent mental health. Indeed, longitudinal studies have shown strong associations of school climate with adolescent mental health (e.g. lower depressive symptoms [DS]) (Hinze et al., 2024; Raniti, Rakesh, Patton, & Sawyer, 2022), and a prior cluster randomized controlled trial (RCT) observed large effects of school climate intervention on adolescent mental health (e.g. *d* = −1.19 on DS [Shinde et al., 2020]). These observations suggest that school climate requires greater research focus to improve adolescent mental health, considering that adolescents spend the majority of their time in schools with increasing rates of enrolment and retention globally (United Nations Educational, Scientific and Cultural Organization, 2017).

The COVID-19 pandemic appeared to cause diverse changes in social lives through public health measures (e.g. restrictions on social contact and recreational activities), which may lead to aggravated adolescent mental health (DeVylder et al., 2024; Hosozawa et al., 2024; Racine et al., 2021). This may suggest that the negative effects of the pandemic differed according to the quality of social environments that individuals felt. However, no study has examined whether the impacts of the pandemic differed according to levels of school climate, which is a major indicator of the quality of adolescent social environments. This is a large knowledge gap to better understand the mechanisms of the negative effects of the pandemic on adolescent mental health and to improve public health measures under future pandemics.

We previously reported the impacts of pandemic on adolescent mental health (i.e. DS [Hosozawa et al., 2024] and psychotic experiences [PEs] [DeVylder et al., 2024]) without considering school climate, using data from the Tokyo Teen Cohort (TTC), an ongoing study following 3171 adolescents in metropolitan Tokyo (Ando et al., 2019). COVID-19 occurred midway through fourth wave (age 16) data collection (DeVylder et al., 2024; Hosozawa et al., 2024), providing a unique opportunity to understand the effects of COVID-19 in a sophisticated design; the data from TTC can compare the outcomes of the *pre-pandemic* group with that of the *during-pandemic* group while accounting for levels of the outcomes at previous three waves (age 10, 12, 14). To date, the TTC is the only adolescent cohort experiencing such a natural experimental situation. With this TTC dataset, the current study aimed to examine whether the pandemic effects differed according to adolescent self-rated school climate.

## Methods

### Study design and procedure

The TTC is an ongoing population-based cohort study (Ando et al., 2019), consisting of random sampling of adolescents born during 2002–2004, using the Basic Resident Register of the Tokyo metropolitan area with oversampling of adolescents in lower-income households. Data was collected when the participants were aged 10 (T1: *N* = 3171), 12 (T2: *N* = 3007), 14 (T3: *N* = 2667), and 16 (T4: *N* = 2614). The TTC study protocol was approved by the Ethics Committees of the Tokyo Metropolitan Institute of Medical Science (#12–35), the University of Tokyo (#10057), and SOKENDAI (Graduate University for Advanced Studies; #2012002). Written informed consent was obtained from all parents. The authors assert that all procedures contributing to this work comply with the ethical standards of the relevant national and institutional committees on human experimentation and with the Helsinki Declaration of 1975, as revised in 2008.

### Measurements

#### School climate

School climate was self-rated using an adapted version of the 28-item Beyond Blue School Climate Questionnaire (Shinde et al., 2018, 2020) (score range: 0–28) at T4. This questionnaire assesses the following components of school climate: ‘Supportive Teacher Relationships’ (e.g. ‘In this school, teachers and students really trust one another’), ‘Student Belonging’ (e.g. ‘Most other students accept me as I am’), ‘Student Participation in School Activities and Decisions’ (e.g. ‘Students have a say in decisions affecting them at this school’), and ‘Personal Commitment to Academic Values’ (e.g. ‘Doing well in school is important to me’). Higher scores indicate a better-perceived school climate. The original Beyond Blue School Climate Questionnaire does not define a specific time frame for most items, except for one item: ‘Thinking of my teachers this term, I really like’ (in Japan, a term typically lasts 3–4 months). Consistent with this, we did not specify a time frame for the adapted version used in this study. Additionally, we assessed school climate-related statuses using original questions (e.g. ‘Does your homeroom teacher help your classmates who are not feeling well?’) at T1. Detailed questions are shown in online Supplementary Tables S1 and S2.

#### Depressive symptoms (DS)

DS were self-rated using the Short Mood and Feelings Questionnaire (Thapar & McGuffin, 1998) (score range: 0–26) at T1–T4, with higher scores indicating worsening symptoms. Scores for participants with one missing item (missing rate: 0.98–1.83% of the total sample across T1–T4) were imputed using person-mean scores (Eyre et al., 2021; Hosozawa et al., 2024). For the analysis of DS, our previous study restricted the study sample to adolescents with valid responses to DS at T4 and at least at T1–T3 (*N* = 2034) (Hosozawa et al., 2024). Among them, the proportion of missing data on school climate was low (*N* = 148: 7.3%).

#### Psychotic experiences (PEs)

PEs were self-rated using the items derived from the schizophrenia section of the Diagnostic Interview Schedule for Children (Costello, Edelbrock, & Costello, 1985), with an added question on visual hallucinations (‘Have you ever seen things that other people could not see?’). Each item was scored on a three-point scale: no (0); maybe (0.5); and yes, definitely (1), and an aggregated score was calculated (score range: 0–5), which was treated as a continuous variable for statistical analysis (DeVylder et al., 2024). For the analysis of PEs, our previous study restricted the study sample based on the same criterion as DS (*N* = 1935). Among them, the proportion of missing data on school climate was low (*N* = 54: 2.8%).

### Statistical analysis

**Figure 1.**
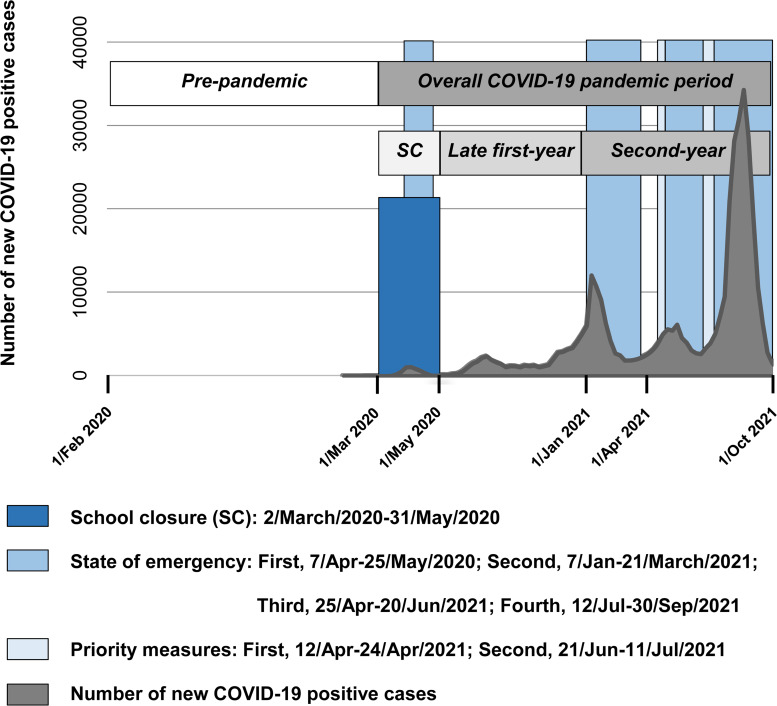
Phases of the COVID-19 and related measures in Tokyo. During the nationwide school closure period (Tokyo Metropolitan Government, 2023), nearly all schools across Japan were closed (Ministry of Education, Culture, Sports, Science and Technology, 2020a, 2020b, 2020c), and students were not permitted to attend in-person classes. During this time, schools provided textbooks and teaching materials for self-study. A minority of schools also offered digital educational content, such as TV broadcasts (24%) and digital textbooks (29%) (Ministry of Education, Culture, Sports, Science and Technology, 2020d). However, only a small proportion (5%) of schools implemented online education via video conferencing platforms (Ministry of Education, Culture, Sports, Science and Technology, 2020d). The nationwide school closure was imposed only once during the pandemic period in Japan (Tokyo Metropolitan Government, 2023).

The COVID-19 pandemic occurred midway through T4 (March 2020), naturally dividing participants into *pre-pandemic* and *during-pandemic* groups (coded post-hoc based on the assessment date) (Fig. 1). Our previous studies reported the negative effects of the COVID-19 pandemic on DS and PEs by analyzing differences in each outcome between participants in the *pre-pandemic* and *during-pandemic* groups at T4 while controlling for within-person changes in each outcome across surveys using mixed-effect models (DeVylder et al., 2024; Hosozawa et al., 2024). The models were adjusted for chronological age at T4 and slope of elapsed time (in months) from the start of the COVID-19 pandemic in Japan (calculated based on the assessment date), without considering the influence of school climate. Based on the natural trajectories of each outcome, we adjusted for linear and quadratic slopes in the analysis of DS (Kwong et al., 2019) and for linear slope in the analysis of PEs (Staines et al., 2022).

To examine whether the effects of the pandemic differed according to adolescent self-rated school climate, we further included the school climate score (continuous) and its interaction with the indicator of the *during-pandemic* group in the model. Both variables were multiplied by another indicator of the T4 survey, because the pandemic and its interaction affected DS and PEs only at T4 (DeVylder et al., 2024; Hosozawa et al., 2024). Additionally, to examine whether this effect modification of school climate differed by phases of the pandemic, the *during-pandemic* period (consisting of the *overall COVID-19 pandemic period*) was divided into three periods (*school closure/late first-year/second-year*). Mixed-effects models are maximum likelihood-based analyses and robust for missing data in outcomes under the assumption of ‘missing at random’ (Bell & Fairclough, 2014). The significance level was set at *α* = 0.05. All analyses were conducted using R version 4.3.2, with lmerTest package.

## Results

The school climate score and its related statuses (assessed at T4 and T1, respectively) did not show significant differences between the *pre-pandemic* and *during-pandemic* groups in both datasets for DS (Table 1 and online Supplementary Table S1) and PEs (Table 1 and online Supplementary Table S2). Scores for DS and PEs did not significantly differ between the two groups across all surveys, except for the DS score at T4, when the pandemic occurred (Table 1). Given the non-normal distribution of these continuous outcomes, we calculated the median and mean absolute deviation (online Supplementary Table S3), which are more robust measures for non-normally distributed data and potential outliers. We compared the scores between the *pre-pandemic* and *during-pandemic* groups using a non-parametric test (i.e. Mann–Whitney *U* test) (online Supplementary Table S3). The significance of group differences remained consistent, with only the DS score at T4 showing a significant difference between the two groups. There were slight significant differences in age at T4 between the *pre-pandemi*c and *during-pandemic* groups (0.4 and 0.3 years in the datasets for DS and PEs, respectively). Detailed information on the sample characteristics is available elsewhere (DeVylder et al., 2024; Hosozawa et al., 2024). In brief, demographic variables assessed, including household income and recreational drug use such as cannabis, were not significantly different between the groups at all timepoints. On the other hand, there were small significant differences in proportions (less than 4%) for low household income throughout the surveys between samples included and excluded in the dataset for DS. Moreover, the age at T4 was slightly higher (0.2 years) among the samples excluded compared to those included in the dataset for PEs.

**Table 1.** Characteristics of study participants included in the current study by timing of age 16 survey (pre-pandemic group or during-pandemic group)

	For DS (*N* = 2034)				
	Pre-pandemic (*n* = 960, 47.1%)		During-pandemic (*n* = 1074, 52.9%)		
Variable	*n*	(%), mean (s.d.)	*n*	(%), mean (s.d.)	*p* value ^a^
Age at T4	960	16.6 (0.3)	1074	17.0 (0.4)	< 0.001
Sex					
Boy	505	(52.6)	559	(52.0)	0.84
Girl	455	(47.4)	515	(48.0)	
DS score at T1	913	4.7 (4.6)	1041	4.5 (4.3)	0.24
DS score at T2	879	3.9 (4.4)	912	3.7 (4.4)	0.37
DS score at T3	866	3.1 (4.5)	881	3.0 (4.7)	0.72
DS score at T4	960	3.3 (4.9)	1074	4.2 (5.5)	< 0.001
School climate score at T4	930	22.0 (5.3)	956	22.4 (5.3)	0.11

	For PEs (*N* = 1935)				
	Pre-pandemic (*n* = 951, 49.1%)		During-pandemic (*n* = 984, 50.9%)		
	*n*	(%), mean (s.d.)	*n*	(%), mean (s.d.)	
Age at T4	951	16.6 (0.3)	984	16.9 (0.3)	< 0.001
Sex					
Boy	502	(52.8)	511	(51.9)	0.74
Girl	449	(47.2)	473	(48.1)	
PEs score at T1	913	0.91 (0.92)	956	0.86 (0.91)	0.20
PEs score at T2	859	0.59 (0.85)	841	0.59 (0.84)	0.91
PEs score at T3	851	0.36 (0.67)	818	0.37 (0.73)	0.78
PEs score at T4	951	0.21 (0.52)	984	0.23 (0.59)	0.38
School climate score at T4	928	22.0 (5.3)	953	22.4 (5.3)	0.08

DS, depressive symptoms; PEs, psychotic experiences; T1, age 10 survey; T2, age 12 survey; T3, age 14 survey; T4, age 16 survey.

a *p* values were derived from *t* test for continuous variables and from chi-square test for dichotomous variables.

**Figure 2.**
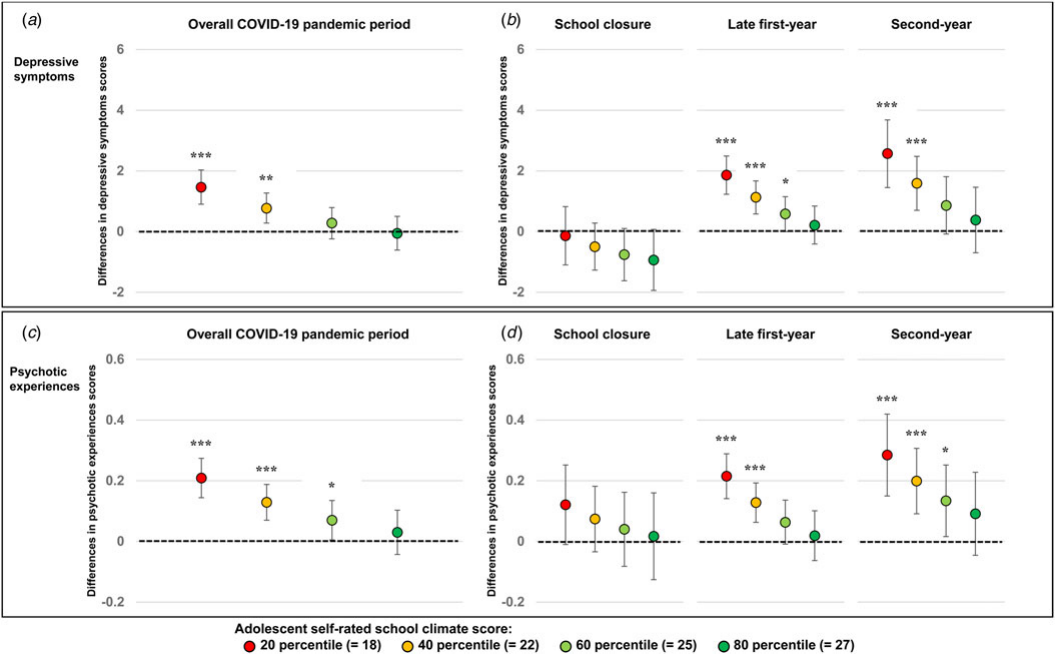
Visualization of differences in the effects of the COVID-19 pandemic on adolescent mental health by adolescent self-rated school climate. *p < 0.05, **p < 0.01, ***p < 0.001. The effects of the COVID-19 on depressive symptoms during (a) *Overall COVID-19 pandemic period* and (b) during *School closure*, *Late first-year*, and *Second-year*. The effects of the COVID-19 on psychotic experiences during (c) *Overall COVID-19 pandemic period* and (d) during *School closure*, *Late first-year*, and *Second-year*. Error bars represent 95% confidence intervals.

For the analysis of DS, the interaction effect between *during-pandemic* and school climate was significant (unstandardized coefficient [*B*] = −0.166, 95% confidence interval [CI] −0.225 to −0.107) (Table 2). Significant interactions were also observed during *late first-year* (*B* = −0.183, 95% CI −0.254 to −0.112) and *second-year* (*B* = −0.243, 95% CI −0.387 to −0.100) (Table 3). The negative effects of the COVID-19 pandemic were not significant on DS among adolescents who had high (approximately 80 percentile or more) school climate score at any phase of the pandemic (Fig. 2).

**Table 2.** Moderation effects of school climate on associations between the COVID-19 pandemic and adolescent mental health during *overall COVID-19 pandemic period*

	Outcome (School climate)
	*B* (95% CI)
Explanatory Variable	For DS (*N* = 2034)
Intercept	3.659 (3.019 to 4.298)***
Time (in year, linear) ^a^	0.321 (0.047 to 0.595)*
Time (in year, quadratic) ^a^	0.074 (0.043 to 0.105)***
Age at T4^b^	−0.081 (−0.450 to 0.289)
Pre-pandemic (*n* = 960)^c^	(Reference)
During-pandemic (*n* = 1074) ^d^	4.419 (2.994 to 5.845)***
School climate	−0.015 (−0.036 to 0.006)
During-pandemic & School climate interaction	−0.166 (−0.225 to −0.107)***

	For PEs (*N* = 1935)
Intercept	0.048 (−0.023 to 0.120)
Time (in year, linear) ^a^	−0.118 (−0.127 to −0.108)***
Age at T4^b^	0.071 (−0.002 to 0.144)
Pre-pandemic (*n* = 951)^c^	(Reference)
During-pandemic (*n* = 984)^d^	0.567 (0.387 to 0.747)***
School climate	0.002 (−0.0005 to 0.0044)
During-pandemic & School climate interaction	−0.020 (−0.028 to −0.012)***

*B*, Unstandardized coefficient; CI, confidence interval; DS, depressive symptoms; PEs, psychotic experiences; T1, age 10 survey; T2, age 12 survey; T3, age 14 survey; T4, age 16 survey.

a Centered at the start time of the COVID-19 pandemic in Japan.

b Centered at age 16.

c Number of participants in pre-pandemic group.

d Number of participants in during-pandemic group.

*p < 0.05; **p < 0.01; ***p < 0.001.

**Table 3.** Moderation effects of school climate on associations between the COVID-19 pandemic and adolescent mental health, during *school closure*, *late first-year*, and *second-year* of the pandemic

	Outcome (School climate)
	*B* (95% CI)
Explanatory Variable	For DS (*N* = 2034)
Intercept	3.418 (2.768 to 4.067)***
Time (in year, linear) ^a^	0.187 (−0.095 to 0.469)
Time (in year, quadratic)^a^	0.059 (0.027 to 0.091)***
Age at T4^b^	−0.089 (−0.459 to 0.280)
Pre-pandemic (*n* = 960)^c^	(Reference)
School closure (*n* = 162)^d^	1.446 (−1.613 to 4.504)
Late first-year (*n* = 678)^d^	5.153 (3.467 to 6.839)***
Second-year (*n* = 234)^d^	6.941 (3.569 to 10.31)***
School climate	−0.007 (−0.029 to 0.014)
School climate & Pre-pandemic interaction	(Reference)
School climate & School closure interaction^c^	−0.088 (−0.221 to 0.045)
School climate & Late first-year^c^	−0.183 (−0.254 to −0.112)***
School climate & Second-year^c^	−0.243 (−0.387 to −0.100)***

	For PEs (*N* = 1935)
Intercept	0.053 (−0.019 to 0.125)
Time (in year, linear)^a^	−0.118 (−0.128 to −0.108)***
Age at T4^b^	0.063 (−0.010 to 0.137)
Pre-pandemic (*n* = 951)^c^	(Reference)
School closure (*n* = 158)^d^	0.328 (−0.101 to 0.757)
Late first-year (*n* = 650)^d^	0.608 (0.390 to 0.825)***
Second-year (*n* = 176)^d^	0.673 (0.240 to 1.105)**
School climate	0.002 (−0.0005 to 0.0044)
School climate & Pre-pandemic interaction	(Reference)
School climate & School closure interaction^c^	−0.012 (−0.030 to 0.007)
School climate & Late first-year^c^	−0.022 (−0.031 to −0.012)***
School climate & Second-year^c^	−0.022 (−0.040 to −0.003)*

*B*, Unstandardized coefficient; CI, confidence interval; DS, depressive symptoms; PEs, psychotic experiences; T1, age 10 survey; T2, age 12 survey; T3, age 14 survey; T4, age 16 survey.

a Centered at the start time of the COVID-19 pandemic in Japan.

b Centered at age 16.

c Number of participants in pre-pandemic group.

d Number of participants in during-pandemic group according to the ‘stage of pandemic.’.

*p < 0.05; **p < 0.01; ***p < 0.001.

Similar results were obtained for the analysis of PEs. The interaction effect between *during-pandemic* and school climate was significant (*B* = −0.020, 95% CI −0.028 to −0.012) (Table 2). Significant interactions were also observed during *late first-year* (*B* = −0.022, 95% CI −0.031 to −0.012) and *second-year* (*B* = −0.022, 95% CI −0.040 to −0.003) (Table 3). The negative effects of the COVID-19 pandemic were not significant on PEs among adolescents who had high (approximately 80 percentile or more) school climate score at any phase of the pandemic (Fig. 2).

## Discussion

This is the first study to examine whether the impacts of the COVID-19 pandemic on DS and PEs among adolescents differed according to the levels of adolescent self-rated school climate, which is considered to be a proximal social determinant of adolescent mental health. We found that the negative effects of the pandemic both on DS and PEs were significantly lower among adolescents who experienced a better school climate, and that the negative effects of the pandemic were not significant among adolescents who scored high school climate (80 percentile or more). These results may suggest that quality of social environments that individuals feel can buffer the impact of actual diverse changes in social lives, extending the suggested mechanism that diverse social changes caused by the pandemic lead to aggravated adolescent mental health (DeVylder et al., 2024; Hosozawa et al., 2024; Racine et al., 2021).

School climate score itself (Table 1) and prior levels of its related statuses (online Supplementary Tables S1 and S2) were not significantly different between the *pre-pandemic* and *during-pandemic* groups. These results may suggest that supportive school social environments (e.g. better interpersonal relationships among students with teachers/peers) (Shinde et al., 2018) can be maintained even when the pandemic caused diverse changes in social lives; in schools, for example, many school events/activities (e.g. school festivals, field trips, and talking in lunch time) were cancelled/restricted due to public health measures (e.g. restrictions on social contact and recreational activities) (Takaku, Shobako, & Nakata, 2024). Improving school climate in daily basis is considered to be crucial to improve adolescent mental health (Shinde et al., 2018, 2020; Yamaguchi et al., 2023), and such improved supportive environments can also play protective roles for difficult situations such as the pandemic.

School climate can be improved through daily activities. For example, a large cluster RCT before the COVID-19 pandemic reported a promising effect of a school climate intervention program that included whole-school activities (e.g. a letterbox system providing a platform for students to raise concerns, complaints, and suggestions), group-level activities (e.g. forming peer groups to assist in organizing various activities such as skit presentations and role plays during school assemblies), and individual-level activities (e.g. one-on-one counseling for students), which resulted in a significant improvement in school climate (*d* = 2.23) (Shinde et al., 2018). These activities, which focus on creating supportive school environments, also significantly reduced adolescent DS (*d* = −1.19), an effect size much larger than that associated with commonly used individual psychotherapy methods, such as cognitive behavioral therapy (*d* = −0.21) (Werner-Seidler et al., 2021, meta-analysis). Thus, school climate presents a vital target for interventions aimed at improving adolescent mental health, including DS (Singla, Shinde, Patton, & Patel, 2021). However, evidence for interventions specifically targeting school climate remains limited. Further research is needed to establish a robust evidence base regarding the effectiveness of school climate interventions on adolescent mental health across diverse populations.

The current study had several limitations. First, although TTC dataset was a unique to examine the impacts of the COVID-19 with sophisticated design, caution may be required to interpret the results as causal effects. For example, we did not directly assess COVID-19 infection, and thus, we cannot rule out a direct biological pathway from COVID-19 infection to adolescent mental health. However, the infection rate among adolescents (ages 10–19) during the pandemic period at T4 (from March 2020 to July 2021) was low, at less than 1.2%, in Tokyo (Bureau of Public Health, Tokyo Metropolitan Government, 2022; Statistics Division, Bureau of General Affairs, Tokyo Metropolitan Government, 2020, 2021), where participants in the TTC were recruited. Furthermore, several studies have reported negligible or small impacts of COVID-19 infection on mental health (e.g. anxiety/depression symptoms [Odds ratio = 1.08] [Klaser et al., 2021] and PEs [Odds ratio = 1.36] [Oh et al., 2021a, 2021b]). Collectively, these findings indicate that COVID-19 infection likely had a minimal effect on our results. Second, the during-pandemic group was slightly older than the pre-pandemic group. However, the age differences were minimal (0.4 and 0.3 years in the datasets for DS and PEs, respectively), and age was not significantly associated with outcome levels in the analyses for both DS and PEs (Tables 2 and 3). Consequently, these age differences are unlikely to have introduced substantial bias in the estimates. Third, dropout rates were significantly higher among adolescents who were older (0.2 years) at T4 and from lower-income households (less than 4% difference). However, these differences were small and may have not led to much bias in the estimates. Finally, the TTC sample mostly consisted of participants of Asian ethnicity living in Japanese metropolitan areas, limiting generalizability of the results to other populations.

## Conclusions

The impacts of the COVID-19 on mental health significantly attenuated among adolescents who felt better school climate, suggesting that supportive school social environments can play a protective role for the negative effects of diverse social changes caused by the pandemic among adolescents. School climate interventions should be conducted to improve quality of social environments in schools, which are central places in adolescent daily lives.

## Supporting information

Yamaguchi et al. supplementary material Yamaguchi et al. supplementary material

## Supplementary material

The supplementary material for this article can be found at https://doi.org/10.1017/S0033291724003192

## References

[ref1] Ando, S., Nishida, A., Yamasaki, S., Koike, S., Morimoto, Y., Hoshino, A., Scientific, T. T. C., … Team, D. C. (2019). Cohort profile: The Tokyo Teen Cohort study (TTC). International Journal of Epidemiology, 48(5), 1414–1414g. doi: 10.1093/ije/dyz033 30879075 PMC6857749

[ref2] Bell, M. L., & Fairclough, D. L. (2014). Practical and statistical issues in missing data for longitudinal patient-reported outcomes. Statistical Methods in Medical Research, 23(5), 440–459. doi: 10.1177/0962280213476378 23427225

[ref3] Bureau of Public Health, Tokyo Metropolitan Government. (2022). Details of new coronavirus positive patient announcement in Tokyo. Available from: https://catalog.data.metro.tokyo.lg.jp/dataset/t000055d0000000367 [last accessed 16 October 2024].

[ref4] Costello, E. J., Edelbrock, C. S., & Costello, A. J. (1985). Validity of the NIMH diagnostic interview schedule for children: A comparison between psychiatric and pediatric referrals. Journal of Abnormal Child Psychology, 13(4), 579–595. doi: 10.1007/BF00923143 4078188

[ref5] DeVylder, J., Yamaguchi, S., Hosozawa, M., Yamasaki, S., Ando, S., Miyashita, M., … Nishida, A. (2024). Adolescent psychotic experiences before and during the COVID-19 pandemic: A prospective cohort study. Journal of Child Psychology and Psychiatry, 65(6), 776–784. doi: 10.1111/jcpp.13907 37953733

[ref6] Eyre, O., Bevan Jones, R., Agha, S. S., Wootton, R. E., Thapar, A. K., Stergiakouli, E., … Riglin, L. (2021). Validation of the short mood and feelings questionnaire in young adulthood. Journal of Affective Disorders, 294, 883–888. doi: 10.1016/j.jad.2021.07.090 34375216 PMC8411664

[ref7] Hinze, V., Montero-Marin, J., Blakemore, S.-J., Byford, S., Dalgleish, T., Degli Esposti, M., … Kuyken, W. (2024). Student- and school-level factors associated with mental health and well-being in early adolescence. Journal of the American Academy of Child and Adolescent Psychiatry, 63(2), 266–282. doi: 10.1016/j.jaac.2023.10.004 37866473 PMC10935542

[ref8] Hosozawa, M., Ando, S., Yamaguchi, S., Yamasaki, S., DeVylder, J., Miyashita, M., … Nishida, A. (2024). Sex differences in adolescent depression trajectory before and into the second year of COVID-19 pandemic. Journal of the American Academy of Child and Adolescent Psychiatry, 63(5), 539–548. doi: 10.1016/j.jaac.2023.08.016 37805069

[ref9] Klaser, K., Thompson, E. J., Nguyen, L. H., Sudre, C. H., Antonelli, M., Murray, B., … Steves, C. J. (2021). Anxiety and depression symptoms after COVID-19 infection: Results from the COVID symptom study app. *Journal of Neurology*, Neurosurgery and Psychiatry, 92(12), 1254–1258. doi: 10.1136/jnnp-2021-327565 34583944 PMC8599635

[ref10] Kwong, A. S. F., Manley, D., Timpson, N. J., Pearson, R. M., Heron, J., Sallis, H., … Leckie, G. (2019). Identifying critical points of trajectories of depressive symptoms from childhood to young adulthood. Journal of Youth and Adolescence, 48(4), 815–827. doi: 10.1007/s10964-018-0976-5 30671716 PMC6441403

[ref11] Ministry of Education. (2020a). Regarding the implementation status of temporary school closures, examples of initiatives (March 19 2020) (Japanese). Available from: https://www.mext.go.jp/content/20200319-mxt_kouhou02-000004520_1.pdf [last accessed 16 October 2024].

[ref12] Ministry of Education. (2020b). Regarding the implementation status of temporary school closures to prevent novel coronavirus infections. (April 22 2020) (Japanese) Available from: https://www.mext.go.jp/content/20200424-mxt_kouhou01-000006590_1.pdf [last accessed 16 October 2024].

[ref13] Ministry of Education. (2020c). Regarding the implementation status of temporary school closures to prevent novel coronavirus infections (May 11 2020) (Japanese). Available from: https://www.mext.go.jp/content/20200513-mxt_kouhou02-000006590_2.pdf [last accessed 16 October 2024].

[ref14] Ministry of Education. (2020d). Regarding the status of efforts on learning guidance at public schools related to temporary school closures to prevent novel coronavirus infections. (Japanese) Available from: https://www.mext.go.jp/content/20200421-mxt_kouhou01-000006590_1.pdf [last accessed 16 October 2024].

[ref15] Oh, H., Goehring, J., Rajkumar, R., Besecker, M., Zhou, S., & DeVylder, J. E. (2021a). COVID-19 dimensions and psychotic experiences among US college students: Findings from the healthy mind study 2020. Schizophrenia Research, 237, 148–152. doi: 10.1016/j.schres.2021.09.003 34534946 PMC8438539

[ref16] Oh, H., Schiffman, J., Marsh, J., Zhou, S., Koyanagi, A., & DeVylder, J. (2021b). COVID-19 infection and psychotic experiences: Findings from the healthy minds study 2020. Biological Psychiatry Global Open Science, 1(4), 310–316. doi: 10.1016/j.bpsgos.2021.05.005 34877564 PMC8639180

[ref17] Racine, N., McArthur, B. A., Cooke, J. E., Eirich, R., Zhu, J., & Madigan, S. (2021). Global prevalence of depressive and anxiety symptoms in children and adolescents during COVID-19: A meta-analysis. JAMA Pediatrics, 175(11), 1142–1150. doi: 10.1001/jamapediatrics.2021.2482 34369987 PMC8353576

[ref18] Raniti, M., Rakesh, D., Patton, G. C., & Sawyer, S. M. (2022). The role of school connectedness in the prevention of youth depression and anxiety: A systematic review with youth consultation. BMC Public Health, 22(1), 2152. doi: 10.1186/s12889-022-14364-6 36424575 PMC9694921

[ref19] Shinde, S., Weiss, H. A., Varghese, B., Khandeparkar, P., Pereira, B., Sharma, A., … Patel, V. (2018). Promoting school climate and health outcomes with the SEHER multi-component secondary school intervention in Bihar, India: A cluster-randomised controlled trial. The Lancet, 392(10163), 2465–2477. doi: 10.1016/S0140-6736(18)31615-5 30473365

[ref20] Shinde, S., Weiss, H. A., Khandeparkar, P., Pereira, B., Sharma, A., Gupta, R., … Patel, V. (2020). A multicomponent secondary school health promotion intervention and adolescent health: An extension of the SEHER cluster randomised controlled trial in Bihar, India. PLOS Medicine, 17(2), e1003021. doi: 10.1371/journal.pmed.1003021 32045409 PMC7012396

[ref21] Singla, D. R., Shinde, S., Patton, G., & Patel, V. (2021). The mediating effect of school climate on adolescent mental health: Findings from a randomized controlled trial of a school-wide intervention. Journal of Adolescent Health, 69(1), 90–99. doi: 10.1016/j.jadohealth.2020.09.030 33127241

[ref22] Staines, L., Healy, C., Coughlan, H., Clarke, M., Kelleher, I., Cotter, D., & Cannon, M. (2022). Psychotic experiences in the general population, a review; definition, risk factors, outcomes and interventions. Psychological Medicine, 52(15), 1–12. doi: 10.1017/S0033291722002550 PMC9772919 36004805

[ref23] Statistics Division, Bureau of General Affairs, Tokyo Metropolitan Government. (2020). Tokyo Households and Population by District and Age Group by Basic Resident Ledger, January 2020. Available from: https://www.toukei.metro.tokyo.lg.jp/juukiy/2020/jy20000001.htm [last accessed 16 October 2024].

[ref24] Statistics Division, Bureau of General Affairs, Tokyo Metropolitan Government. (2021). Tokyo Households and Population by District and Age Group by Basic Resident Ledger, January 2021. Available from: https://www.toukei.metro.tokyo.lg.jp/juukiy/2021/jy21000001.htm [last accessed 16 October 2024].

[ref25] Takaku, R., Shobako, N., & Nakata, T. (2024). Three years of COVID-19-related school restrictions and mental health of children and adolescents in Japan. Scientific Reports, 14(1), 16707. doi: 10.1038/s41598-024-67138-y 39030262 PMC11271618

[ref26] Thapar, A., & McGuffin, P. (1998). Validity of the shortened Mood and Feelings Questionnaire in a community sample of children and adolescents: A preliminary research note. Psychiatry Research, 81(2), 259–268. doi: 10.1016/s0165-1781(98)00073-0 9858042

[ref27] Tokyo Metropolitan Government. (2023). Initiatives taken by the Tokyo Metropolitan Government for COVID-19 response (Revised June 2 2023). Available from: https://www.seisakukikaku.metro.tokyo.lg.jp/documents/d/seisakukikaku/e0602 [last accessed 16 October 2024].

[ref28] United Nations Educational, Scientific and Cultural Organization. (2017). Reducing global poverty through universal primary and secondary education. Retrieved from http://unesdoc.unesco.org/images/0025/002503/250392E.pdf [last accessed 8 August 2024].

[ref29] Werner-Seidler, A., Spanos, S., Calear, A. L., Perry, Y., Torok, M., O'Dea, B., … Newby, J. M. (2021). School-based depression and anxiety prevention programs: An updated systematic review and meta-analysis. Clinical Psychology Review, 89, 102079. doi: 10.1016/j.cpr.2021.102079 34571372

[ref30] Yamaguchi, S., Ando, S., Miyashita, M., Usami, S., Yamasaki, S., Endo, K., … Nishida, A. (2023). Longitudinal relationships between help-seeking intentions and depressive symptoms in adolescents. Journal of Adolescent Health, 73(6), 1061–1067. doi: 10.1016/j.jadohealth.2023.06.033 37665304

