# Supplementary material for: Protective role of school climate for impacts of COVID-19 on depressive symptoms and psychotic experiences among adolescents: a population-based cohort study
Source: Psychol Med. 2024 Dec 20;54(16):4878–85. doi: 10.1017/S0033291724003192 (PMC11769899; doi:10.1017/S0033291724003192)
Supplement: Yamaguchi et al. supplementary material [file S0033291724003192sup001.docx]

| Table S1. School climate related statuses among study participants included for analysis of depressive symptoms by timing of age 16 survey (pre-pandemic group or during-pandemic group) | | | | | |
| --- | --- | --- | --- | --- | --- |
|  | For DS (N = 2,034) | | | |  |
|  | Pre-pandemic  (n = 960, 47.1%) | | During-pandemic  (n = 1,074, 52.9%) | |  |
| School climate status at age 10 survey | n | (%) | n | (%) | p-value ^c)^ |
| Do you like your school? |  |  |  |  |  |
| Very much/A little | 904 | (95.5) | 1013 | (95.0) | 0.73 |
| Not at all | 43 | (4.5) | 53 | (5.0) |  |
| Do you like your homeroom teacher? ^a)^ |  |  |  |  |  |
| Very much/A little | 843 | (88.4) | 964 | (90.3) | 0.19 |
| Not at all | 111 | (11.6) | 104 | (9.7) |  |
| Do your classmates do things they should not do or cause trouble in class |  |  |  |  |  |
| Always/almost always | 393 | (41.2) | 398 | (37.3) | 0.08 |
| Sometimes/Not at all | 562 | (58.8) | 669 | (62.7) |  |
| Do your classmates help other classmates who are not feeling well? |  |  |  |  |  |
| Always/almost always | 568 | (59.7) | 647 | (60.9) | 0.61 |
| Sometimes/Not at all | 384 | (40.3) | 416 | (39.1) |  |
| Does your homeroom teacher help your classmates who are not feeling well? ^a)^ |  |  |  |  |  |
| Always/almost always | 769 | (80.9) | 871 | (81.9) | 0.64 |
| Sometimes/Not at all | 181 | (19.1) | 193 | (18.1) |  |
| Help-seeking intention from classmates ^b)^ |  |  |  |  |  |
| Yes | 571 | (60.9) | 645 | (61.1) | 0.96 |
| No | 367 | (39.1) | 411 | (38.9) |  |
| Help-seeking intention from school teacher ^b)^ |  |  |  |  |  |
| Yes | 472 | (50.3) | 519 | (49.1) | 0.63 |
| No | 466 | (49.7) | 537 | (50.9) |  |
| DS: depressive symptoms  a) In the Japanese school system, students are assigned to a "homeroom" class, which is managed by a homeroom teacher. Each homeroom class typically consists of around 30-40 students, and the homeroom teacher plays a central role in the students' school life. In addition to teaching their subject, homeroom teachers are responsible for various aspects of student life, including taking attendance, communicating daily school schedules, providing academic and career guidance, and supervising class activities, especially during school events such as festivals. Most teachers in Japan serve as homeroom teachers for a major part of their careers.  b) Help-seeking intention was assessed as follows. First, participants read a vignette describing a boy with depressive symptoms as the following description: “For the last several weeks, Taro has been feeling unusually sad. He is tired all the time and has trouble sleeping at night. Taro doesn’t feel like eating and has lost weight. He can’t keep his mind on his studies, and his grades have dropped. He puts off making any decisions, and even day-to-day tasks, such as studying and extracurricular activities, seem too much for him. His parents and teachers are very concerned about him.” After reading the vignette, the participants were asked “Who would you seek help from if you were in the same situation as the boy (Taro)?” The response options were as follows: Friends, Family member, Relative, School teacher, School counselor, Doctor, Online bulletin board. Participants can endorse multiple options.  c) p-values were derived from chi-square test | | | | | |
| Table S2. School climate related statuses among study participants included for analysis of psychotic experiences by timing of age 16 survey (pre-pandemic group or during-pandemic group) | | | | | |
|  | For PEs (N = 1,935) | | | |  |
|  | Pre-pandemic  (n = 951, 49.1%) | | During-pandemic  (n = 984, 50.9%) | |  |
| School climate status at age 10 survey | n | (%) | n | (%) | p-value ^c)^ |
| Do you like your school? |  |  |  |  |  |
| Very much/A little | 898 | (95.7) | 928 | (95.0) | 0.50 |
| Not at all | 40 | (4.3) | 49 | (5.0) |  |
| Do you like your homeroom teacher? ^a)^ |  |  |  |  |  |
| Very much/A little | 837 | (88.7) | 885 | (90.5) | 0.22 |
| Not at all | 107 | (11.3) | 93 | (9.5) |  |
| Do your classmates do things they should not do or cause trouble in class |  |  |  |  |  |
| Always/almost always | 388 | (41.0) | 366 | (37.5) | 0.12 |
| Sometimes/Not at all | 558 | (59.0) | 611 | (62.5) |  |
| Do your classmates help other classmates who are not feeling well? |  |  |  |  |  |
| Always/almost always | 563 | (59.8) | 591 | (60.7) | 0.72 |
| Sometimes/Not at all | 379 | (40.2) | 383 | (39.3) |  |
| Does your homeroom teacher help your classmates who are not feeling well? ^a)^ |  |  |  |  |  |
| Always/almost always | 763 | (81.2) | 799 | (82.0) | 0.67 |
| Sometimes/Not at all | 177 | (18.8) | 175 | (18.0) |  |
| Help-seeking intention from classmates ^b)^ |  |  |  |  |  |
| Yes | 568 | (61.2) | 590 | (61.1) | 0.99 |
| No | 360 | (38.8) | 376 | (38.9) |  |
| Help-seeking intention from school teacher ^b)^ |  |  |  |  |  |
| Yes | 471 | (50.8) | 476 | (49.3) | 0.55 |
| No | 457 | (49.2) | 490 | (50.7) |  |
| PEs: psychotic experiences  a) Role of homeroom teacher is described in the footnote of Table S1  b) Assessment of help-seeking intention is described in the footnote of Table S1  c) p-values were derived from chi-square test | | | | | |

| Table S3. Median and mean absolute deviation in mental health status scores among study participants included in the current study by timing of age 16 survey (pre-pandemic group or during-pandemic group) | | | | | |
| --- | --- | --- | --- | --- | --- |
|  | For DS (N = 2,034) | | | |  |
|  | Pre-pandemic  (n = 960, 47.1%) | | During-pandemic  (n = 1,074, 52.9%) | |  |
| Variable | n | median (MAD) | n | mean (MAD) | p-value ^a)^ |
| DS score at T1 | 913 | 3 (3.6) | 1,041 | 3 (3.4) | 0.45 |
| DS score at T2 | 879 | 2 (3.3) | 912 | 2 (3.4) | 0.15 |
| DS score at T3 | 866 | 1 (3.3) | 881 | 1 (3.4) | 0.24 |
| DS score at T4 | 960 | 1 (3.6) | 1,074 | 2 (4.1) | < 0.001 |
|  | For PEs (N = 1,935) | | | |  |
|  | Pre-pandemic  (n = 951, 49.1%) | | During-pandemic  (n = 984, 50.9%) | |  |
|  | n | median (MAD) | n | median (MAD) |  |
| PEs score at T1 | 913 | 0.50 (0.74) | 956 | 0.50 (0.74) | 0.15 |
| PEs score at T2 | 859 | 0.00 (0.65) | 841 | 0.00 (0.67) | 0.72 |
| PEs score at T3 | 851 | 0.00 (0.49) | 818 | 0.00 (0.52) | 0.58 |
| PEs score at T4 | 951 | 0.00 (0.33) | 984 | 0.00 (0.37) | 0.83 |
| DS: depressive symptoms; MAD: mean absolute deviation around mean; PEs: psychotic experiences; T1: age 10 survey; T2: age 12 survey; T3: age 14 survey; T4: age 16 survey  a) p-values were derived from Mann-Whitney U test | | | | | |
